# ELSA recommendations for minimally invasive surgery during a community spread pandemic: a centered approach in Asia from widespread to recovery phases

**DOI:** 10.1007/s00464-020-07618-0

**Published:** 2020-05-11

**Authors:** Asim Shabbir, Raj K. Menon, Jyoti Somani, Jimmy B. Y. So, Mahir Ozman, Philip W. Y. Chiu, Davide Lomanto

**Affiliations:** 1 Department of Surgery, National University Hospital, Singapore, Singapore; 2 Division of Infectious Diseases, Department of Medicine, National University Hospital, Singapore, Singapore; 3 Department of Surgery, Yong Loo Lin School of Medicine, National University of Singapore, Singapore, Singapore; 4 Department of Surgery, School of Medicine, Istinye University, Istanbul, Turkey; 5 Division of Upper GI & Metabolic Surgery, Department of Surgery, Faculty of Medicine, The Chinese University of Hong Kong, Hong Kong, Hong Kong

**Keywords:** COVID-19, MIS, Surgery, Laparoscopic surgery, Guidelines, Recommendations

## Abstract

**Background:**

The COVID-19 pandemic has resulted in significant changes to surgical practice across the worlds. Some countries are seeing a tailing down of cases, while others are still having persistent and sustained community spread. These evolving disease patterns call for a customized and dynamic approach to the selection, screening, planning, and for the conduct of surgery for these patients.

**Methods:**

The current literature and various international society guidelines were reviewed and a set of recommendations were drafted. These were circulated to the Governors of the Endoscopic and Laparoscopic Surgeons of Asia (ELSA) for expert comments and discussion. The results of these were compiled and are presented in this paper.

**Results:**

The recommendations include guidance for selection and screening of patients in times of active community spread, limited community spread, during times of sporadic cases or recovery and the transition between phases. Personal protective equipment requirements are also reviewed for each phase as minimum requirements. Capability management for the re-opening of services is also discussed. The choice between open and laparoscopic surgery is patient based, and the relative advantages of laparoscopic surgery with regard to complications, and respiratory recovery after major surgery has to be weighed against the lack of safety data for laparoscopic surgery in COVID-19 positive patients. We provide recommendations on the operating room set up and conduct of general surgery. If laparoscopic surgery is to be performed, we describe circuit modifications to assist in reducing plume generation and aerosolization.

**Conclusion:**

The COVID-19 pandemic requires every surgical unit to have clear guidelines to ensure both patient and staff safety. These guidelines may assist in providing guidance to units developing their own protocols. A judicious approach must be adopted as surgical units look to re-open services as the pandemic evolves.

The COVID-19 pandemic continues to grip the globe and outbreaks across continents and countries appears to be in various phases of containment and mitigation. Countries like China have seen an early surge followed by a sustained recovery, while others in Asia, like Singapore, after controlling the curve are now seeing surging numbers. There remain concerns about middle income Asian countries with limited screening and containment resources, resulting in a poor understanding of the extent of disease. This could result in the hypothetical but real concern of not only a resurgence over time but also that these countries could become reservoirs of ongoing infection. There are diverse economic conditions in Asia. Affluent countries seem to be able to absorb the financial shock of diverting resources to testing and treating COVID-19 patients, while putting most of the elective or non-emergency care on hold. Other Asian countries are having difficulty diverting resources from standard care and may be unable to hold back the provision of much need emergency and elective surgical care for long. The COVID-19 pandemic across Asia has interrupted the delivery of not only elective surgical care, but also has had a distinct impact on the use of minimally invasive surgery (MIS). MIS has benefits of early recovery, shorter length of stay and equitable cost in certain scenarios and these value-adds have promulgated to wider adaptation of MIS across Asia. The general approach of reducing or stopping elective surgery to support emergency care and fulfill the needs of COVID-19 patients is very reasonable where there is wide community spread with limited system capacity. However, we should bear in mind that across Asia, many surgical institutions may not be called upon to support the COVID-19 efforts due to centralization of tertiary care. These surgical institutions may want to continue some level of non-emergent surgical care when there is limited community spread. There is a need during this COVID-19 pandemic for a surgical practice guideline in Asia that could be tailored to the needs of specific countries and their communities depending on the spread of COVID-19 and their local resources.

The recommendations below (Table 1) are an extract from the current scientific evidence and are endorsed by experts in the field (Governors of the Endoscopic and Laparoscopic Surgeons of Asia. These overarching principles do not supersede clinical judgment and the ultimate responsibility of care, resource utilization and safety is owned by the specialist.

**Table 1:** Strategies for patient selection for surgery during the COVID-19 outbreak

Surgical management/pandemic response state	Active community spread/high ICU utilization/health system overwhelmed	Limited community spread/ICU capacity maintained	Isolated/mostly imported cases	No active alerts
Case selection—elective cases	Limit all elective cases including oncology to maintain capacity for intensive care and high dependency beds; Delay elective surgery for all COVID-19 suspect/positive cases	Allow oncology, trauma, obstetric cases. Limit non-urgent cases which require intensive care and high dependency beds. Stop day surgery, short stay surgery and reduce operating volume to create capacity for staff training. Delay elective surgery for all COVID-19 suspect/positive cases	Proceed with all elective surgery. Consider delaying non-urgent, non-oncology cases to expedite staff training. Delay elective surgery for all COVID-19 suspect/positive cases	As per normal running
Screening—elective cases	In countries with strong contact tracing capability—naso/oropharyngeal swab pre-operatively for elective cases for patients WITH acute respiratory illness, pneumonia, or patients with a positive travel/contact history. Consideration of swabbing all elective patients in areas with poor contact tracing pre-operatively	Naso/oropharyngeal swab pre-operatively or delay surgery for elective cases for patients WITH acute respiratory illness, pneumonia, or patients with a positive travel/contact history	Naso/oropharyngeal swab pre-operatively or delay surgery for elective cases for patients WITH acute respiratory illness, pneumonia, or patients with a positive travel/contact history	As per normal running
Case selection—emergency cases	Non-surgical management preferred—treatment with antibiotics and interventional radiology preferentially	Non-surgical management considered for mild disease—treatment with antibiotics and interventional radiology preferentially	Continue routine management of emergency cases	As per normal running
Screening—emergency cases	Presume high priority cases with no accurate symptom/contact history as COVID-19 suspect/positive	Presume high priority cases with no accurate symptom/contact history as COVID-19 suspect/positive	Presume high priority cases with no accurate symptom/contact history as COVID-19 suspect/positive	As per normal running
PPE requirements	Full PPE for all elective and emergency cases (N-95 masks, gloves, gowns and goggles/face shields). Consideration of PAPR for aerosol generating procedures and head and neck surgery	Full PPE for COVID-19 positive/suspect cases. Surgical mask and face shields at minimum for all non-COVID-19 positive/suspect cases. Consideration of PAPR for aerosol generating procedures and head and neck surgery	Full PPE for COVID-19 positive/suspect cases. Surgical mask and face shields at minimum for all non-COVID-19 positive/suspect cases	As per normal running
Reopening strategy—in sequence as per capability	Do not restart services	Restart full oncology, trauma and obstetric elective services	Restart day surgery, short stay cases	Restart all elective surgery

The recommendation for proceeding with MIS listed here assume two scenarios of community spread—wide spread versus a limited community spread.

## Wide community spread vs limited spread/recovery phase

Where there is wide community spread of COVID-19, deferring elective surgery is highly recommended. The Iranian series by Aminian et al. [1] speaks about COVID-19 complicating the perioperative course with diagnostic challenges and a high potential post-operative fatality. The other approach would be to screen everyone planned for elective surgery; however, this is a very resource and labor intense process and even then, may not be fool-proof as there are varying reports on the sensitivity, specificity, positive predictive value and negative predictive values of the available COVID-19 tests.

In communities, where there is very limited spread of disease or they if are in a recovery phase, elective surgery is suggested to be phased with opening of essential services such as surgical oncology and elective trauma work followed by more benign procedures which if postponed too long can lead to more severe complications, such as cholecystectomies in a patient with gallstone pancreatitis. Finally, all surgical services can revert to normal with the advent of a vaccine or effective therapy. This phased approach allows for a cautious introduction of surgical services while keeping health care workers safe, surgical disease, and COVID-19 at check. It will safe guard patient needs and prevent huge backlogs that would overwhelm the health care systems in Asia and may potentially result in unwanted outcomes in the form of morbidity to mortality.

In limited community spread areas, patients should be assessed for travel history, and reviewed for presence of respiratory symptoms like fever, cough, blocked or running nose, sore throat, shortness of breath and / or gastrointestinal symptoms like, diarrhea, abdominal pain, myalgia and fatigue. In addition, contact history with a suspected or confirmed COVID-19 case should also be reviewed. If either clinical or contact history is suspicious, the patient should be treated as a suspect/positive case and prevailing national guidelines should be followed in provision of care. If the clinical and contact history is not significant, screening for COVID-19 can be waived.

While some guidelines suggest maximal conservative management of surgical emergencies e.g. acute appendicitis and acute cholecystitis [2], *ELSA recommends to weigh the risk of failure of conservative management, complications arising as a result of delayed intervention and the actual effectiveness of conservative management given the potential increased length of stay, increased anxiety, and the usage of valuable bed space, in particular in the high dependency and intensive care units.*

## Selection of patients for minimally invasive surgery

There is no evidence to suggest for or against laparoscopic surgery versus open surgery. The overarching principle would be to provide a safe, optimal, efficient care that is proportionate with the available manpower and infrastructure resources.

The indications for MIS surgery during COVID-19 time do not change. The ISDE recommends postponing transthoracic esophagectomy until virus status is confirmed negative given the risk of pulmonary complications after one-lung ventilation being a major concern [3]. However, open surgery and in particular upper abdominal extensive surgical procedures increase the risk of pulmonary complications and these patients may benefit from MIS technique [4].

## Aerosolization in surgery

All energy devices whether, electrocautery or ultrasonic in nature will produce surgical smoke (plume) when used on tissue and this plume is aerosolized. Aerosolization during laparoscopy is held as the most common reason for holding back laparoscopic surgery during COVID-19 outbreak. However, surgical plume results from tissue desiccation and thus the use of an electrocautery even in open surgery is potentially hazardous. Studies have shown that activated virus like, HIV and Papilloma virus can be found in surgical smoke that is blown away by the CO2 in laparoscopy and thus potentially infective [5, 6]. Also, hepatitis B virus has previously been demonstrated to be present in surgical smoke from HBV positive patients [7]. Erring on the side of caution, due to limited data on the use of laparoscopy in COVID-19 patients, many societies have cautioned against the use of laparoscopy [8, 9]. Li et al. in their study had shown that for the same energy device activated for 10 min during traditional open surgery versus laparoscopic surgery, the particle concentration of the smoke in laparoscopic surgery was significantly higher owing to low gas mobility in the pneumoperitoneum [10, 11].

It is also known that the low temperature aerosol arising from ultrasonic scalpels cannot effectively deactivate the cellular components of a virus. Another matter of concern is that the virus may concentrate in the gastrointestinal tract and surgical plume can thus be a source of infection even though the respiratory system is sealed by a closed and filtered respiratory circuit [12]. Mitigation strategies for aerosol formation and handling will be detailed in the recommendations below.

## Practical guidelines for general surgery

The decision to operate, how to operate and when to operate must be made by the most senior person in the team and this preferably should be a specialist consultant considering the risk/benefits of each treatment.

1. A detailed informed consent and explanation should be offered to the patient with regard to the implications of having a COVID-19 infection on both the patient and staff
2. Operating after hours should be avoided and the most appropriate skilled person as chosen by the team lead should perform the surgery
3. Limit the number of staff in the operating room to minimize exposure
4. Intubate and extubate within the operating room itself
5. Allow for a 5-min pause during intubation and extubation, with only the anesthetists and assistants, donning full PPE, to be in the operating room. This ensures that at least two full gas exchanges of the OR are taken and enhances safety in the very unlikely chance that the surgeons are operating on an undiagnosed COVID-19 case [13]
6. Trainee’s participation in surgery is best not done as an operating surgeon given that it will delay surgery and may potentially risk complications and safety issues may arise [11]
7. The operating rooms ideally should be one as descried by Ti et al. [14]. An OR with a negative pressure environment located at a corner of the operating complex, and with a separate access, is designated for all confirmed (or suspected) COVID-19 cases. Only the ante room and anesthesia induction rooms have negative atmospheric pressures. But for countries with limited resources, an alternate OR complexes separated from the main OR can be identified for COVID-19 patients, thus avoiding contamination of other ORs.
8. Judicious use of PPE which includes a N-95 mask for high risk cases, impervious gown, double gloves, shields or goggles are recommended along with non-perforated shoes or rubber boots [15].
9. No definitive evidence that PAPR reduces likelihood of viral transmission for potential airborne infections [16].
10. Use regional anesthesia where possible to reduce intubation and general anesthesia related risk
11. Effective communication between the operating team, anesthetist and support staff is of paramount in helping to reduce operating time and improving safety.
12. Surgical plume needs to be removed effectively. During open surgery consider use of electrocautery pencil with suction.
13. Team changes will be required for prolonged procedures in full PPE [17].
14. Adequate disposable of waste at the end of surgery.
15. During shifting of suspected COVID-19 patient to an outside recovery area or intensive care unit, handing over to a minimum number of transport personnel who are waiting outside the OR should be considered [18].
16. Personal protective equipment during transport should be not be the same as worn during the procedure [18].
17. Continued education on the occupation hazard and updates on new research will help real time adjustment of protocols.

## Endo-laparoscopic surgery during widespread and localized/recovery phase

Laparoscopic surgery may proceed provided patients are selected carefully, gas is managed well along with surgical plume as shown by the published surgical experience during COVID-19 outbreak [11].

For a patient with active COVID-19, the use of laparoscopy should be carefully considered, because of the lack of safety data for staff.

A. Individualize the approach and timing of surgery by balancing the risk of disease progression with the risk of surgery and aerosolization
B. Trocar insertion site incision should be sized such that it admits the trocar but does not allow air leak. Purse-string suture or disposable trocar with skin blocking system should be used
C. Disposable trocars should be used as reusable trocars may contain virus load after cleansing
D. Pneumoperitoneum creation should be undertaken using a technique that one is most familiar with. The Veress needle is a good choice where expertise exists. An optical trocar to gain access can be used too, otherwise, Hasson’s technique with a good seal with prevention of air leak is important
E. Pneumoperitoneum should be maintained at a lower pressure (10-12 mm of Hg) and low flow rate of gas insufflation
F. All pneumoperitoneum should be safely evacuated via a filtration system before trocar’s removal, port site closure, specimen extraction or conversion to open surgery
G. Avoid using two-way pneumoperitoneum insufflators to prevent pathogen colonization of circulating aerosol in pneumoperitoneum circuit or the insufflator
H. Prevent creation of plume by
  - Minimizing energy device usage and avoid its prolonged activation
  - For endo-laparoscopic electrocautery set power at low
  - Avoid long dissecting time at same spot using the device to reduce surgical plume
  - Suction frequently to avoid accumulation of plume in the intra-abdominal cavity
  - Keep instruments clean of blood and tissues and operating surface dry to minimize plume formation.
I. Smoke evacuation
  - Passive or Active Filtration Should be utilized.
  - Use of commercially available smoke evacuating system is encouraged. The specification of the filtration is an important consideration in COVID-19 as the virus itself measures approximately 0.12 microns and systems like RapidVac^TM^smoke evacuator system by Medtronic uses an ultra-low particulate air filter (ULPA) that can remove 99.99% of the particles that are 0.12microns or more in diameter.
  - Where a commercially available system can’t be obtained due to cost or licensing issues a DIY technique that uses a draining tube connected to a ventilating port on the abdominal side and then to an underwater seal containing a virucidal solution like CIDEX® that connects to suction device can be used.
  - Standard electrostatic filters used for ventilation offer 99.99% effective protection against HBV and HCV which have a diameter of 42 nm and 30–60 nm, respectively. SARS-CoV-2 has a larger diameter of 70–90 nm therefore according to an accepted publication of Mintz et al. [19] the filter can be connected via standard tubing to the trocar evacuation port to constitute an evacuation and filtering system which evacuates the generated smoke, as well as filters the potential viral load to ensure surgical staff safety without the need for active suction.
J. The use of more complex integrated systems like the IES3 Erbe, Conmed Airseal system®,Megadyne Mega Vac Plus or MiniVac, S-PILOT Karl Storz are recommended where resources are available
K. The use of disposable instruments where possible is advisable. Reusing disposables, is not approved due to concerns over sterility and the clinical consequence of residual viral load [20, 21]
L. Reusable instrument’s reprocessing durability must be considered as they are subject to wear and tear. The use of reusable instruments may be wary about quality reducing over time, insufficient sterility and equipment failure [22]
M. Watch out for sharp instruments and handle with care as a prick may damage protective equipment
N. Use surgical drains only if strictly necessary
O. Teach and practice safe surgery

## References

[1] Aminian A, Safari S, Razeghian-Jahromi A, Ghorbani M, Delaney CP (2020). COVID-19 outbreak and surgical practice: unexpected fatality in perioperative period. Ann Surg.

[2] American College of Surgeons (2020) COVID 19: elective case triage guidelines for surgical care. https://www.facs.org/covid-19/clinical-guidance/elective-case. Assessed 25 Apr 2020

[3] PWY Chiu, C Hassan, CY Hon, G Antonelli, P Sharma (2020) Management of upper-GI endoscopy and surgery in COVID-19 outbreak. https://isde.net/covid19-guidance. Assessed 25 Apr 2020

[4] Walker JL, Piedmonte MR, Spirtos NM, Eisenkop SM, Schlaerth JB, Mannel RS, Spiegel G, Barakat R, Pearl ML (2009). Sharma SK (2009) Laparoscopy compared with laparotomy for comprehensive surgical staging of uterine cancer: Gynecologic Oncology Group Study LAP2. J Clin Oncol.

[5] Hensman C, Baty D, Willis RG (1998). Cuschieri A (1998) Chemical composition of smoke produced by high-frequency electrosurgery in a closed gaseous environment. Surg Endosc.

[6] Johnson GK (1991). Robinson WS (1991) Human immunodeficiency virus-1 (HIV-1) in the vapors of surgical power instruments. J Med Virol.

[7] Kwak HD, Kim SH, Seo YS, Song KJ (2016). Detecting hepatitis B virus in surgical smoke emitted during laparoscopic surgery. Occup Environ Med.

[8] Society of American Gastrointestinal and Endoscopic Surgeons (2020) SAGES and EAES recommendations regarding surgical response to COVID-19 cases. https://www.sages.org/recommendations-surgical-response-covid-19/. Accessed 30 Mar 2020

[9] Association of Upper Gastrointestinal Surgeons (2020) Intercollegiate general surgery guidance on COVID-19. https://www.augis.org/wp-content/uploads/2020/03/intercollegiate-surg-guidance-COVID-19-infographic2.pdf. Accessed 27 Mar 2020

[10] Li CI, Pai JY, Chen CH (2020). Characterization of smoke generated during the use of surgical knife in laparotomy surgeries. J Air Waste Manag Assoc.

[11] Zheng MH, Boni L, Fingerhut A (2020). Minimally invasive surgery and the novel coronavirus outbreak: lessons learned in China and Italy. Ann Surg.

[12] Zhang W, Du RH, Li B, Zheng XS, Yang XL, Hu B, Wang YY, Xiao GF, Yan B, Shi ZL, Zhou P (2020). Molecular and serological investigation of 2019-nCoV infected patients: implication of multiple shedding routes. Emerg Microbes Infect.

[13] Chew MH, Koh FH, Ng KH (2020). A call to arms: a perspective of safe general surgery in Singapore during the COVID-19 pandemic. Singapore Med J.

[14] Ti LK, Ang LS, Foong TW, Ng BSW (2020). What we do when a COVID-19 patient needs an operation: operating room preparation and guidance. Can J Anaesth.

[15] Karaca AS, Ozmen MM, Ucar AD, Yasti AC, Demirer S (2020). General surgery operating room practice in patients with COVID-19. Turk J Surg.

[16] Wong J, Goh QY, Tan Z, Lie SA, Tay YC, Ng SY, Soh CR (2020). Preparing for a COVID-19 pandemic: a review of operating room outbreak response measures in a large tertiary hospital in Singapore. Can J Anesth.

[17] Royal College of Surgeons (2020) Updated general surgery guidance on COVID-19. https://www.rcseng.ac.uk/coronavirus/joint-guidance-for-surgeons-v2/. Accessed 15 Apr 2020

[18] American College of Surgeons (2020) COVID-19: considerations for optimum surgeon protection before, during, and after operation. https://www.facs.org/covid-19/clinical-guidance/surgeon-protection. Assessed 15 Apr 2020

[19] Mintz Y, Arezzo A, Boni L, Chand M, Brodie R, Fingerhut A, Technology Committee of the European Association for Endoscopic Surgery (2020). A low cost, safe and effective method for smoke evacuation in laparoscopic surgery for suspected coronavirus patients. Ann Surg.

[20] Morrison JE, Jacobs VR (2004). Replacement of expensive, disposable instruments with old-fashioned surgical techniques for improved cost-effectiveness in laparoscopic hysterectomy. JSLS.

[21] Brusco JM, Ogg MJ (2010). Health care waste management and environmentally preferable purchasing. AORN J.

[22] Apelgren KN, Blank ML, Slomski CA, Hadjis NS (1994). Reusable instruments are more cost-effective than disposable instruments for laparoscopic cholecystectomy. Surg Endosc.

